# Tumor fat composition predicts response to everolimus and supports low-dose therapy in TSC-associated renal angiomyolipomas: a multicenter prospective study

**DOI:** 10.3389/fonc.2026.1893155

**Published:** 2026-07-22

**Authors:** Ke Gao, Yongfei Duan, Biqi Ren, Boxin Guo, Ruiping Su, Hao Gu, Fulin Chen, Chi Zhang, Guojun Wu, Jianxin Ni

**Affiliations:** 1 Department of Urology, Xi’an People’s Hospital (Xi’an Fourth Hospital), School of Medicine, Northwest University, Xi’an, China; 2 Department of Scientific, Xi’an People’s Hospital (Xi’an Fourth Hospital), School of Medicine, Northwest University, Xi’an, China; 3 Laboratory of Tissue Engineering, College of Life Sciences, Northwest University, Xi’an, Shaanxi, China

**Keywords:** everolimus treatment, low-dose therapy, renal angiomyolipoma, tuberous sclerosis complex, tumor fat composition

## Abstract

**Background and objective:**

Renal angiomyolipomas associated with tuberous sclerosis complex (TSC-RAML) may lead to progressive renal damage and hemorrhage. Everolimus is recommended as first-line therapy, but predictors of treatment response and optimal dosing strategies remain unclear. We aimed to evaluate the efficacy of everolimus and to determine whether tumor fat composition predicts treatment response, as well as whether a reduced-dose regimen provides comparable outcomes.

**Methods:**

In this multicenter prospective cohort study conducted across multiple centers in China, 183 consecutive patients with TSC-RAML were enrolled. Three prespecified, outcome-specific analytical subsets were generated from the overall cohort according to the data required for each analysis: (1) standard-dose everolimus efficacy analysis, (2) CT-based tumor fat-composition analysis, and (3) exploratory dose-comparison analysis. Radiographic evaluations were performed at baseline, 3 months, and 6 months. The primary outcome was the RAML response rate, defined as ≥50% reduction in target tumor volume.

**Key findings and limitations:**

A ≥50% reduction in tumor volume occurred in 44% (34/78) of patients at 3 months and 83% (60/72) at 6 months. Tumor reduction was significantly greater in fat-poor than in fat-rich lesions. No significant difference in response was observed between the standard- and low-dose groups. Oral mucositis was the most common adverse event but occurred less frequently in the low-dose group (P = .002). Tumor regrowth was observed after treatment discontinuation. Limitations include the nonrandomized design, in which dose selection was based on clinical criteria from previously published protocols, unequal group sizes in the dose-comparison analysis, and the open-label nature of treatment administration, which may introduce selection and ascertainment bias.

**Conclusions and clinical implications:**

In this large Chinese cohort of patients with TSC-RAML, everolimus effectively reduces tumor volume, particularly in fat-poor lesions. CT-derived tumor fat composition may help predict treatment response. In this exploratory cohort, low-dose everolimus showed promising short-term activity with potentially improved tolerability, but randomized studies are needed to confirm comparative efficacy.

## Introduction

Tuberous sclerosis complex (TSC) is an autosomal dominant genetic disorder affecting multiple systems throughout the body, including the central nervous system, skin, eyes, oral cavity, and various internal organs (1–3). Its incidence ranges from 1/10,000 to 1/6,000, affecting males and females equally. Most TSC cases are sporadic, whereas approximately one-third of all TSC cases have a family history (4–6). Renal angiomyolipoma (RAML) is the most common renal manifestation of TSC, occurring in up to 80% of the cases (7–9). Compared with sporadic RAML, TSC-RAML is characterised by earlier onset, faster progression, bilaterally multifocal involvement, and a high risk of bleeding. Although not classified as a malignancy with increasing tumour size, complications such as bleeding risk and rupture-induced arterial hypertension make RAML a leading cause of death in adult patients with TSC (10). Loss−of−function mutations are the main cause of TSC, with 80%–85% of the cases harbouring mutations in *TSC1* or *TSC2* (11–13). We previously observed that *TSC2* mutations are more frequently associated with RAML than *TSC1* mutations (14). In addition, patients with *TSC2* mutations tend to exhibit larger AMLs and a higher frequency of other clinical manifestations than those with *TSC1* mutations. This finding aligns with the observations of Paolo et al. (15).

Mammalian target of rapamycin (mTOR) is a conserved serine/threonine protein kinase frequently activated by mutations in *TSC1* or *TSC2* in patients with TSC, which leads to the development of TSC-related benign tumours or hamartomas in multiple systems (16–19). Inhibitors of mTOR, such as everolimus, have a triple effect in patients with TSC: inhibition of tumour cell growth and proliferation, inhibition of tumour nutrient metabolism and inhibition of angiogenesis, ultimately leading to an effective alleviation of TSC-related systemic symptoms. According to the 2021 International TSC Consensus Guidelines (2), everolimus is recommended as the first-line therapy for asymptomatic, growing angiomyolipomas larger than 3 cm. Nevertheless, although the efficacy of everolimus has been demonstrated in multiple clinical trials, including the EXIST-2 study and its long-term extension (20–22), evidence regarding its effectiveness in specific populations, such as Asian cohorts, remains limited, and the predictive value of intrinsic tumor characteristics, including fat content, for treatment response has not yet been clearly defined. The present multicenter study was conducted exclusively in a Chinese population, providing much-needed real-world data on everolimus efficacy and safety in an Asian cohort. Furthermore, investigating lower-dose regimens holds significant clinical relevance for optimizing long-term tolerability and patient compliance. This investigation is timely, as the 2024 ERKNet/ERA consensus statement explicitly suggests 5 mg/day everolimus as a reasonable starting dose for adults with TSC, highlighting the need for prospective data comparing fixed low−dose and standard−dose regimens (23).

In the current multicenter study, we enrolled a large cohort of 183 Chinese patients with TSC-RAML to comprehensively evaluate the short- to intermediate-term efficacy and individualized use of everolimus in clinical practice. Specifically, we aimed to determine whether the baseline fat composition of RAML—quantified by the mean Hounsfield Unit (HU) on computed tomography (CT)—correlates with treatment response, and whether a fixed low-dose regimen (5 mg/day) can achieve comparable efficacy with improved tolerability relative to the standard 10 mg/day dose. These findings provide complementary evidence that may help refine personalized mTOR inhibitor strategies for patients with TSC-RAML.

## Materials and methods

### Patient selection and inclusion

This multicenter study represents a direct extension of our earlier single-center work (14). By expanding the patient cohort, prospectively evaluating tissue-specific responses, and incorporating a predefined low-dose group, the study substantially deepens and refines the previous research. A total of 208 patients with renal AML were screened at Xi’an People ‘s Hospital (the Fourth Hospital of Xi’an), the First Affiliated Hospital and the Second Affiliated Hospital of the Fourth Military Medical University, 183 of whom were enrolled in this study. This study was conducted in accordance with the Declaration of Helsinki (24). All eligible patients were aged ≥18 years with at least one RAML with its longest diameter measuring ≥3 cm and diagnosed as having TSC according to the diagnostic criteria of the 2021 International Consensus Diagnostic Criteria (2).

This project was reviewed and approved by the Ethics Committee of Xi’an People’s Hospital (the Fourth Hospital of Xi’an) (approval number: 20200024) and the First Affiliated Hospital of the Fourth Military Medical University (approval number: KY20183355-1). Written informed consent was obtained from all participants. The institutional review board approved our study protocol, and the data and safety monitoring board reviewed the progress of the trial on a semiannual basis.

### Study design and treatment

**Figure 1:**
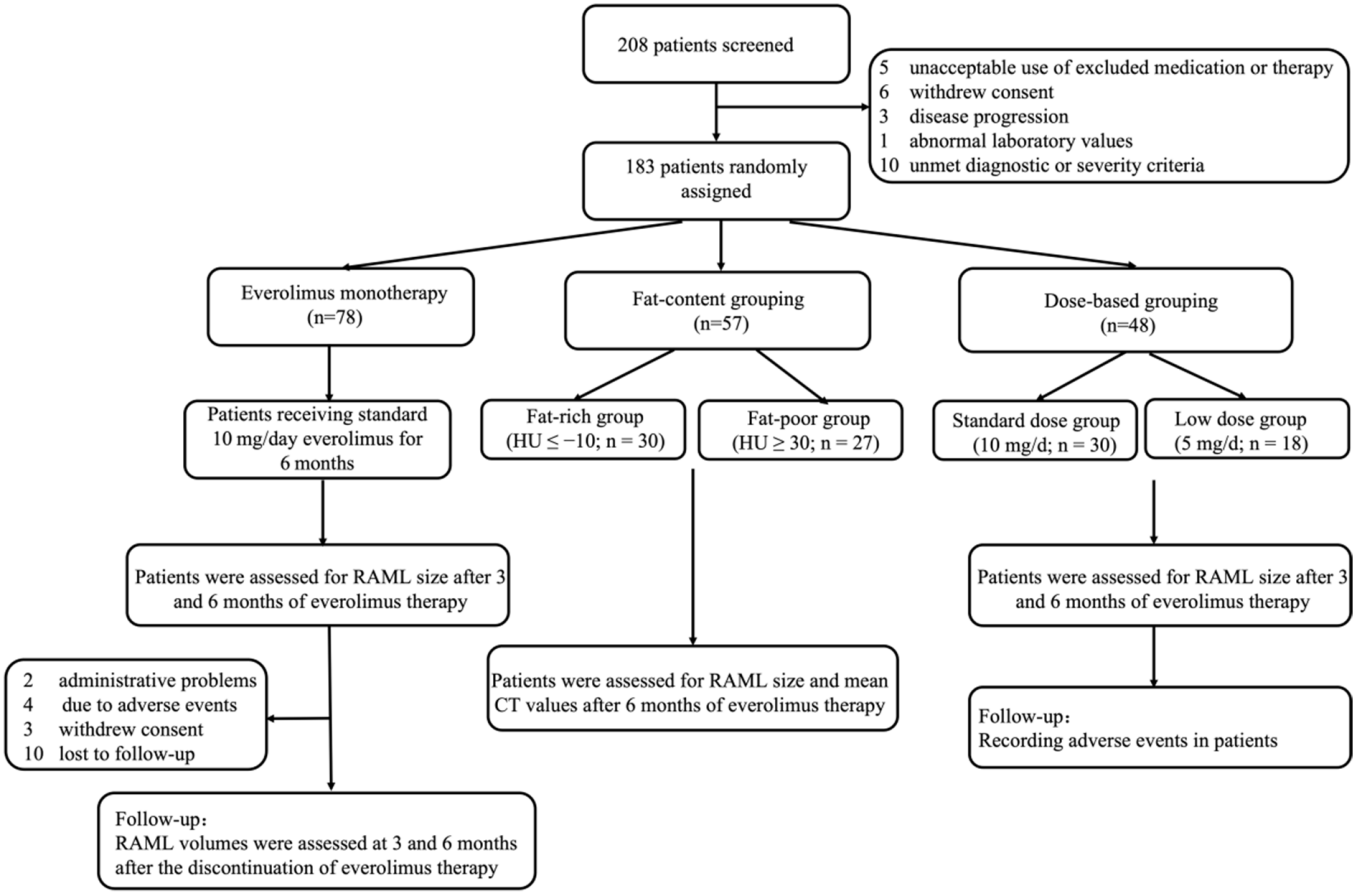
Trial profile and patient inclusion flow.

In this multicenter prospective study, all enrolled patients constituted a single observational cohort. Predefined subgroup analyses were subsequently performed to evaluate treatment efficacy, the influence of tumor fat composition, and differences between standard- and low-dose everolimus regimens. Within this overall cohort, three analytical components were defined: (A) evaluation of everolimus treatment efficacy under the standard dosing regimen (10 mg/day for 6 months), (B) subgroup analysis according to tumor fat composition (fat-rich vs fat-poor RAMLs based on baseline CT-derived mean Hounsfield Unit values), and (C) an exploratory dose-comparison analysis between standard-dose everolimus (10 mg/day) and a low-dose regimen (5 mg/day) (**Figure 1**). The three prespecified analytical subsets together accounted for the entire enrolled cohort. These subsets were defined prospectively according to the data requirements of each analysis and should not be interpreted as randomized or overlapping treatment arms. This approach allowed each research question to be examined in the most appropriate patient group without unnecessarily limiting the sample size available for the other analyses. Specifically, the (A) cohort included patients receiving the standard 10 mg/day regimen in routine clinical practice. The (B) cohort consisted of patients whose baseline CT imaging was technically suitable for quantitative fat-content analysis, defined as unenhanced acquisitions with slice thickness ≤ 5 mm, absence of motion artefact obscuring the target lesion, and complete coverage of the RAML permitting reliable ROI-based mean Hounsfield Unit measurement. Although all prospectively enrolled patients underwent baseline CT as per protocol, a subset of scans performed at referring institutions used heterogeneous acquisition parameters (e.g., non-standard slice thicknesses or contrast-enhanced-only phases) that precluded reproducible HU quantification. These patients were therefore excluded from the fat-composition analysis. The (C) cohort was designed as an exploratory comparison of different fixed-dose regimens; therefore, patients who received either the standard 10 mg/day dose or a low 5 mg/day dose during routine clinical care were included in this analysis. Because the 10 mg/day regimen represents the guideline-recommended standard therapy, a larger proportion of patients received this regimen, resulting in unequal group sizes between the dosing groups. The decision to administer the low-dose (5 mg/day) regimen was made at the treating clinician’s discretion, following the patient selection criteria established by Hatano et al. (25), in which 5 mg/day everolimus was administered to patients with renal dysfunction (serum creatinine ≥ 1.5 mg/dL) or low body weight (body weight < 35 kg), while patients without these conditions received the standard 10 mg/day dose. This practice is also consistent with the 2024 ERKNet/ERA consensus statement, which recommends 5 mg/day everolimus as a reasonable starting dose in adults with TSC, with further adaptation based on side effects and efficacy (23). Consequently, the allocation to dose groups was neither randomized nor blinded, which may introduce selection bias and represents a key limitation of this exploratory comparison.

Central imaging reviewers assessing tumor volume were blinded to the dosing groups; however, treating clinicians were aware of the administered dose to ensure appropriate safety monitoring. The 6−month treatment period was adopted in accordance with the 2024 ERKNet/ERA consensus statement, which recommends a minimal follow−up of 6 months before assessing clinical and radiological response, given that the main tumour−volume reduction effect of mTORC1 inhibitors is typically observed within the first 6–12 months of therapy (23). Everolimus plasma concentrations were measured at 2 weeks to achieve a whole-blood trough concentration of 5–15 ng/mL. Although magnetic resonance imaging (MRI) is the modality recommended by current clinical guidelines, this study primarily employed CT for imaging evaluation, based on the following considerations: (1) As a multicenter study, the use of CT ensured higher accessibility and standardization across participating sites in China, thereby enhancing image quality control and measurement consistency; (2) The grouping criteria for fat content in this study were defined according to CT attenuation values; thus, CT was used throughout the study to maintain consistency in pre- and post-treatment assessments; (3) For the primary endpoint of this study—tumor volume measurement—CT has been validated as a reliable and reproducible modality. We acknowledge the superior sensitivity of MRI in detecting minimal fat components; however, the classification framework of this study was designed to distinguish typical fat-rich and fat-poor RAMLs. Accordingly, MRI was reserved for selected clinical situations, and all volumetric measurements were derived from CT-based analyses. No placebo or untreated control arm was included for ethical reasons—everolimus is established as the standard of care for growing, asymptomatic TSC-associated AMLs, and withholding effective therapy would raise ethical concerns. Therefore, the study focused on within-treatment subgroup comparisons rather than placebo-controlled efficacy testing.

Studies have reported that attenuation values on CT of ≤−30, 30–50, and ≥100 HU are considered to indicate fats (26, 27), muscles (skeletal and smooth) (28), and blood vessels (29), respectively. Because of a lack of consensus on the radiological classification of RAML (30, 31), we established a CT-based classification criterion according to the aforementioned studies: mean CT values ≤ −10 HU indicate fat-rich RAML, whereas mean CT values ≥ 30 HU denote fat-poor RAML. Patients with mean CT values between −10 and 30 HU were excluded. This classification scheme was informed by published imaging studies of TSC-associated RAML. Hatano et al. stratified lesions according to tumor density (lipid-rich lesions with HU ≤ −50 and solid lesions with HU ≥ 30) when evaluating the response to everolimus, supporting the biological relevance of CT attenuation-based classification (32). In addition, Wang et al. demonstrated that baseline CT characteristics, including mean CT attenuation values, were associated with the volumetric response of TSC-RAMLs to mTOR inhibitor therapy (33). Patients with mean CT values between −10 and 30 HU were excluded in the present study for the following reasons: (1) lesions within this range likely represent heterogeneous mixtures of fat, smooth muscle, and vascular components, potentially introducing substantial biological heterogeneity and reducing the contrast between fat-rich and fat-poor phenotypes; (2) exclusion of intermediate-density lesions enabled a clearer evaluation of the relationship between tumor composition and treatment response; and (3) the use of predefined absolute HU thresholds improves reproducibility, facilitates cross-study comparison, and enhances potential clinical applicability compared with sample-dependent classification methods such as median-based grouping.

For each patient, the largest measurable RAML identified on baseline non-contrast CT was selected as the predefined target lesion for longitudinal volumetric assessment. In patients with bilateral or multiple lesions, only this target lesion was included in the primary efficacy analysis and was consistently evaluated at all follow-up examinations by blinded central radiology review. This approach was adopted because the largest lesion is generally the most clinically relevant in TSC-RAML, as lesion size is closely associated with the risk of bleeding and renal complications. In addition, the use of a single predefined target lesion provides a standardized and reproducible method for longitudinal assessment in patients with multifocal and bilateral disease. Tumor response was evaluated according to changes in the volume of the predefined target RAML. The primary efficacy endpoint was the proportion of patients achieving a confirmed response, defined as a ≥50% reduction in target RAML volume relative to baseline. RECIST 1.1 criteria were not applied, as they were developed primarily for malignant solid tumors and are less suitable for benign, frequently multifocal, and irregularly shaped RAMLs, for which three-dimensional volumetric assessment provides a more clinically meaningful evaluation of treatment response. Tumor volume assessments were performed by CT scans at baseline, 3 and 6 months of treatment, and at 3 and 6 months after treatment discontinuation. All scans underwent a central radiological review, and the treatment response rate was defined as the percentage reduction in volume relative to the baseline, calculated as [(V_Pretreatment_ − V_Posttreatment_)/V_Pretreatment_] × 100%, where V_Pretreatment_ and V_Posttreatment_ represent pretreatment and posttreatment volumes, respectively (14).

Adverse events were continuously monitored throughout the study period and graded according to the Common Terminology Criteria for Adverse Events (version 3.0). The assessments were based on patient- or caregiver-reported responses, as well as investigator evaluations. White blood cell and platelet counts, as well as serum hemoglobin, blood urea nitrogen, creatinine, glucose, liver enzyme, bilirubin, lipids, potassium, sodium, total cholesterol, and triglyceride concentrations, were measured routinely. During the study period, concomitant use of potent cytochrome P450 3A4 or P-glycoprotein (PgP) inhibitors or inducers was avoided, and grapefruit juice was not served or used to dilute everolimus.

### Statistical analysis

Data of all treated patients were collated and analysed. Descriptive statistics was used to summarise the baseline characteristics of the study patients, with results presented as a frequency distribution [absolute frequencies (n) and valid percentages (%)]. Statistical significance between groups was determined using the *t* or χ^2^ test. The normality of continuous variables was assessed using the Shapiro-Wilk test. Data that followed a normal distribution are presented as means ± standard deviations (SDs). Additionally, nonparametric tests (i.e. Mann–Whitney *U* test was used for two independent samples and Wilcoxon signed-rank test was used for individual timepoints) were performed; these data are presented as medians with 25 and 75 percentiles. Pearson correlation coefficient was employed to analyse the correlation between age, fat, sex, and everolimus efficacy. In addition, differences in RAML response rates between the standard- and low-dose groups were reported with 95% confidence intervals (CIs). *P* <.05 was considered to indicate statistical significance. All statistical analyses were performed using SPSS (version 26.0.0; IBM).

## Results

### Baseline characteristics of our patients

All patients were prospectively enrolled, and the analysis of tumor fat content was a pre-specified subgroup analysis based on baseline characteristics. The dose-comparison cohort was designed as a prospective nested cohort study within the overall study framework. Among 203 screened participants, 183 fulfilled the inclusion criteria and proceeded to the baseline observation phase. The common clinical features of TSC observed in these patients included subependymal nodules, RAMLs, and dermatological manifestations (e.g. ash-leaf-shaped hypomelanotic macules, facial angiofibromas, Shagreen patches, and ungual fibromas; **Supplementary Figure 1**). Within the overall cohort, different analytical subsets were evaluated to address specific study objectives. For the evaluation of everolimus efficacy under the standard regimen, 78 patients received oral everolimus at 10 mg/day for 6 months. For the fat-composition analysis, 57 patients with baseline CT imaging suitable for quantitative assessment were stratified into fat-rich (n = 30) and fat-poor (n = 27) RAMLs based on mean CT attenuation values. For the exploratory dose-comparison analysis, 48 patients receiving fixed daily doses of everolimus were analyzed, including 30 treated with the standard dose (10 mg/day) and 18 treated with a low dose (5 mg/day). During the study period, 2 patients discontinued participation for administrative reasons, 4 discontinued due to adverse events, 3 withdrew consent, and 20 were lost to follow-up during the study period.

Of the 183 treated patients, 77 (42.08%) were male and 106 (57.92%) were female, indicating a higher proportion of female patients in this treated cohort. The median (range) age of all included patients was 38 (18–68) years. A total of 86 patients (47%) underwent genetic testing, of whom 80% had *TSC2* mutations. Nearly all patients presented with skin lesions at baseline, with 134 (73.22%) patients developing facial angiofibromas. The baseline characteristics of the study population are summarised in **Table 1**.

**Table 1:** Baseline demographic and clinical characteristics.

Characteristics, n (%)	Patients with renal angiomyolipoma (n = 183)
Gender	
Male	77 (42.08)
Female	106 (57.92)
Age (years)	
Median age (range), years	38 (18 – 68)
< 35 years	71 (38.80)
≥ 35 years	112 (61.20)
Genetic testing(n = 86)	
TSC1 pathogenic variant	7 (8.14)
TSC2 pathogenic variant	69 (80.23)
No mutation identified	10 (11.63)
Primitive volume of the largest renal AML	
< 200 cm3	126 (68.85)
≥ 200 cm3	57 (31.15)
Cutaneous manifestations	
Hypomelanotic macules	87 (47.54)
Facial angiofibromas	134 (73.22)
Shagreen patch	67 (36.61)
Ungual fibroma	81 (44.26)
Central nervous system features	
Subependymal giant cell astrocytoma	39 (21.31)
Subependymal nodules	71 (38.80)
Cortical dysplasias	48 (26.23)
Pulmonary features	
Lymphangioleiomyomatosis	69 (37.71)
Oral cavity	
Gingival fibromas	62 (33.88)
Dental enamel pits	69 (37.71)
Other comorbidities	
Liver angiomyolipomas	8 (4.37)
Multiple renal cysts	4 (2.19)
Retinal hamartoma	40 (21.86)

TSC, Tuberous sclerosis complex; AML, Angiolipoma.

Data are presented as n (%) unless otherwise noted. All clinical features align with the major or minor features in the International Tuberous Sclerosis Complex Diagnostic Criteria.

### Everolimus treatment efficacy and RAML progression after treatment discontinuation

In the primary analysis, patients received oral everolimus (10 mg/day) for 6 months; six of these patients were lost follow-up after 3 months of treatment. After 3 months of treatment, the average RAML tumour volume decreased by 46.96% ± 22.25% (range, 4.88%–97.36%; *P* <.001 compared with the baseline). By 6 months of treatment, the median response rate for RAML tumour volume reduction was 74.36% (range, 58.51%–83.91%; *P* <.001 compared with the baseline). These results suggested a rapid reduction in RAML tumour volumes with continued treatment in the short term. However, the differences in the treatment effect with respect to sex, age, and primary volume were nonsignificant (**Table 2**).

**Table 2:** Treatment effect of everolimus in different subgroups.

Characteristics mean ± SD/median (range)	% at 3 months (n = 78)	P-value	% at 6 months (n = 72)	P-value
Gender				
Male	49.64 ± 22.77 (7.84 – 92.61)	.315	78.16 (66.30 – 79.28)	.137a
Female	44.53 ± 21.77 (4.88 – 97.36)		69.92 (56.08 – 76.88)	
Age (years)				
≤ 30 (n = 19)	57.43 (8.11 – 97.36)	.096a	73.25 ± 19.44 (19.25 – 100.00)	.505
30–50 (n = 46)	44.68 (4.88 – 92.61)		67.06 ± 21.79 (10.26 – 100.00)	
> 50 (n = 13)	47.86 (8.36 – 82.91)		65.03 ± 19.36 (29.60 – 84.42)	
Primitive volume				
< 200 cm3 (n = 48)	46.40 ± 22.07 (7.84 – 92.61)	.712	71.17 (58.51 – 82.30)	.368a
≥ 200 cm3 (n = 30)	48.55 ± 23.28 (4.88 – 97.36)		76.53 (59.92 – 85.79)	
Total (n = 78)	46.96 ± 22.25 (4.88 – 97.36)	<.001	74.36 (58.51 – 83.91)	<.001b

^a^ Mann – Whitney U test.

^b^ Wilcoxon signed-rank test.

A *P*-value < 0.05 was considered statistically significant.

**Figure 2:**
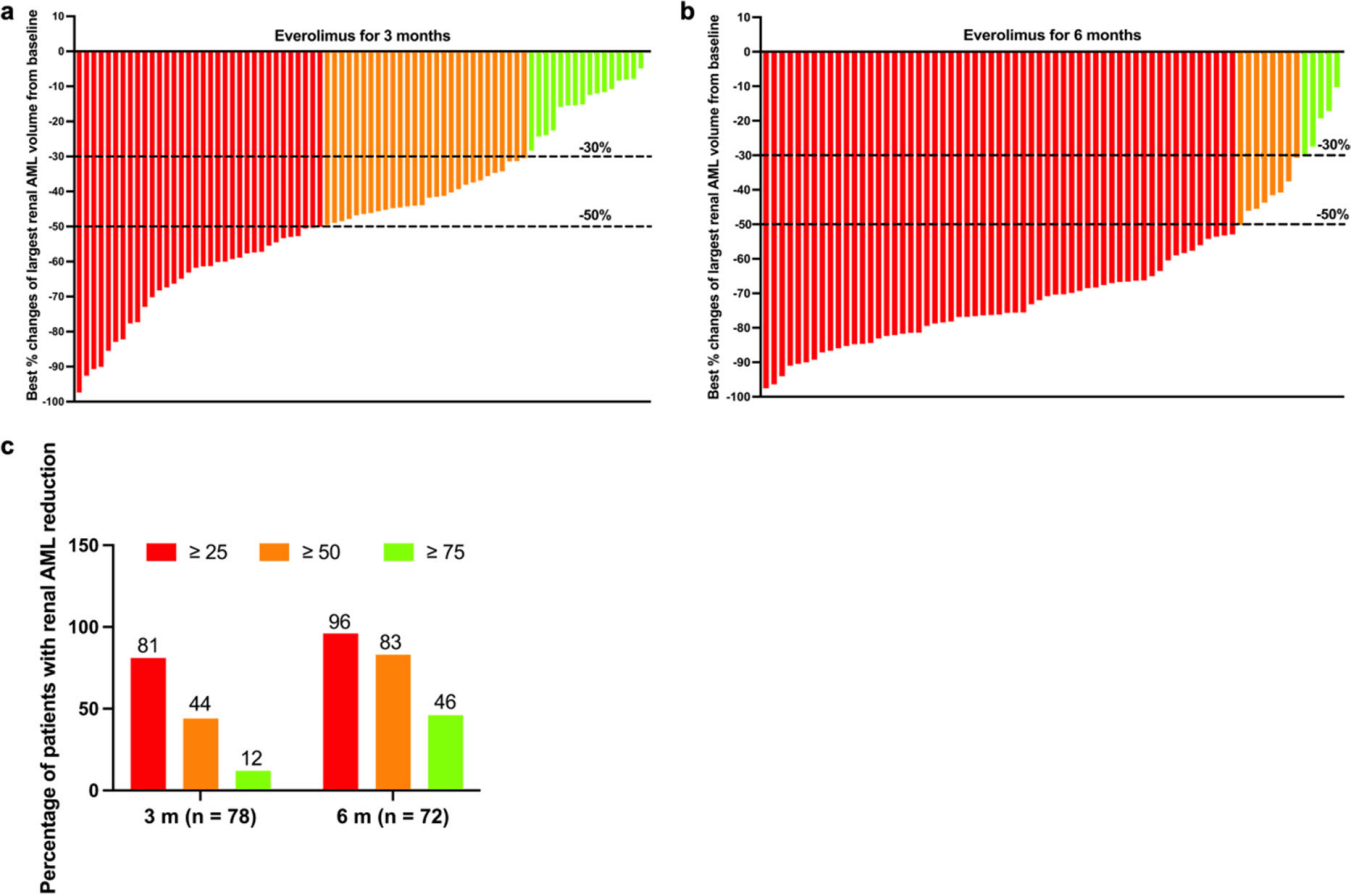
Treatment effect magnitude. **(a, b)** Best percentage changes of largest renal AML volume from baseline levels after 3 **(a)** and 6 **(b)** months of everolimus treatment. Dashed lines indicate clinically relevant cutoffs of ≥30% and ≥50% reductions in RAML tumour volumes compared with the baseline levels. **(c)** Proportion of patients achieving ≥25%, ≥50%, and ≥75% reductions in RAML tumour volumes at different timepoints.

In the complete analysis set, the best percentage reduction in tumor volume from baseline was calculated for each patient based on assessments at 3 and 6 months of treatment (**Figure 2a, b**). According to the central radiology review, target RAML lesion volumes decreased from baseline in almost all patients. At 6 months of treatment, 67 of 72 patients (93.06%) achieved a response rate of >30% in the target RAML lesion, representing a 13.61% increase compared with that at 3 months. The proportions of patients achieving ≥25%, ≥50%, and ≥75% reductions in RAML tumor volume from baseline increased from 81% (63/78), 44% (34/78), and 12% (9/78) at 3 months to 96% (69/72), 83% (60/72), and 46% (33/72) at 6 months (**Figure 2c**). Among the 72 patients who achieved ≥25% tumor volume reduction, 96% (69/72) showed no radiological progression at their final assessment before the end of the study. In addition, CT images visually demonstrated a marked decrease in RAML tumor volume with increasing treatment duration (**Supplementary Figure 2**).

Patients who completed 6 months of everolimus treatment underwent renal CT scans at 3 and 6 months after treatment discontinuation to evaluate changes in tumor volume. As illustrated in **Supplementary Figure 3a**, everolimus treatment for 3–6 months resulted in a marked reduction in tumor size. Compared with baseline, the mean tumor volume decreased by 46.96% (95% CI, 41.94–51.97%) and 67.33% (95% CI, 62.73–71.93%) at 3 and 6 months of therapy, respectively. However, after discontinuation of everolimus, TSC-RAML lesions gradually increased in size. Three months after drug withdrawal, the mean tumor volume had rebounded to 62.5% of the baseline level, and by 6 months it had approached the baseline level. Notably, in 5 patients (7.46%), the RAML volume exceeded the baseline level at 3 months after discontinuation; this number increased to 28 patients (47.46%) at 6 months (**Supplementary Figure 3b**). These findings highlight the occurrence of tumor rebound following everolimus withdrawal and underscore the importance of long-term treatment strategies to achieve sustained disease control in TSC-RAML.

### Effects of everolimus on volumes and densities of RAMLs with varying fat content

Within the overall cohort, 77 patients had baseline CT scans suitable for quantitative analysis. According to the predefined HU thresholds, 30 lesions were classified as fat-rich and 27 as fat-poor. The remaining 20 lesions (26.0% of evaluable cases) had mean CT attenuation values between −10 and 30 HU and were categorized as intermediate-density lesions; these were excluded from the fat-composition analysis to minimize biological heterogeneity and maximize discrimination between the two predefined phenotypes.

Changes in tumour volume and mean CT attenuation before and after treatment are summarized in **Table 3**.

**Table 3:** Change in volume and mean CT value pre- and post- treatment for RAML.

Variables	Fat-rich group (n = 30)	Fat-poor group (n = 27)	P-value
Baseline volume (cm3) Median (25 to 75%)	51.14 (20.25 – 125.49)	46.51 (27.65 – 80.49)	.330
Post-treatment volume (cm3) Median (25 to 75%)	43.12 (15.56 – 96.36)	12.66 (9.20 – 17.41)	<.001
Baseline mean CT value (HU) Mean ± SD	-42.67 ± 18.87	49.48 ± 10.08	<.001
Post-treatment mean CT value (HU) Mean ± SD	-45.00 ± 17.47	34.89 ± 7.39	<.001

A *P*-value < 0.05 was considered statistically significant.

**Figure 3:**
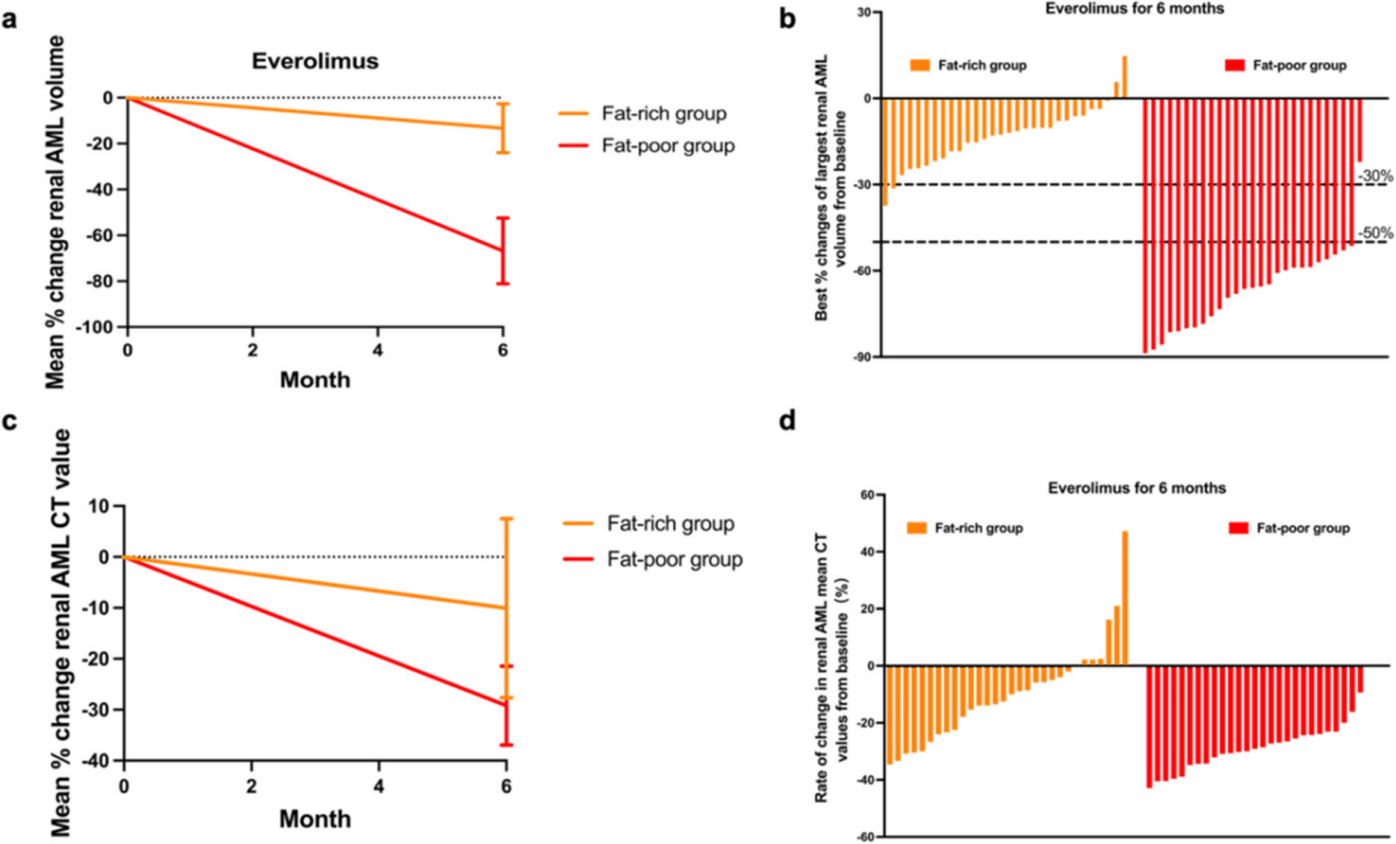
Effects of everolimus on TSC-RAML with varying fat content. **(a)** Mean volume changes in RAMLs following everolimus treatment. **(b)** Best percentage change from baseline in tumor volume of fat-rich and fat-poor RAMLs after 6 months of everolimus treatment. **(c)** Mean CT value change in RAMLs after everolimus treatment. **(d)** Rate of changes in mean CT value from baseline levels in fat-rich and -poor RAML tumours.

Everolimus effectively reduced tumour volumes, but the response varied based on the RAML fat content. The post-treatment tumor volume was significantly larger in the fat-rich RAML group than in the fat-poor RAML group [median 43.12 (15.56–96.36) cm³ vs. 12.66 (9.20–17.41) cm³, *P* <.001], indicating a weaker response to everolimus in fat-rich lesions (**Table 3** and **Figure 3a**). In the fat-rich RAML group, two patients demonstrated an increase in the RAML tumour volume from the baseline: 6.67% (2/30) of patients achieved a ≥30% reduction, but none achieved a ≥ 50% reduction. In contrast, all fat-poor RAML group patients exhibited a reduction in RAML tumour volume; 96.30% (26/27) demonstrated a ≥30% reduction in RAML tumour volume, and an identical proportion of the patients exhibited a ≥ 50% reduction (**Figure 3b**). Moreover, a multivariate analysis of age, fat content, and sex revealed that fat content was the only factor significantly associated with achieving a ≥50% reduction in RAML tumour volume after everolimus treatment (0.965; *P* <.001) (**Supplementary Table 1**). Taken together, these findings demonstrated that everolimus exerted differential effects on RAMLs depending on their fat content, with fat-poor RAML exhibiting a significantly greater response to treatment in terms of volume reduction.

We then analysed the mean CT values of RAML in both groups. At baseline, the mean CT value of tumours was significantly higher in the fat-poor RAML group than in the fat-rich RAML group (49.48 ± 10.08 vs.−42.67 ± 18.87; *P* <.001). After 6 months of treatment, the mean CT value decreased in both groups, with a more considerable reduction observed in the fat-poor RAML group (**Table 3**; **Figure 3c**). We quantified these changes by defining the mean CT value reduction rate as the percentage change in the mean CT value of the tumour relative to its baseline value. Individual patient analyses showed that in the fatty RAML group, the mean CT value increased in 6 patients, remained unchanged in 1 patient, and decreased to varying degrees in the remaining 23 patients. In contrast, the mean CT value decreased in all fat-poor RAML group patients (**Figure 3d**). Thus, everolimus treatment may reduce RAML density, particularly in patients with fat-poor RAMLs.

### Effects of low-dosage everolimus treatment on TSC-RAML tumors

**Figure 4:**
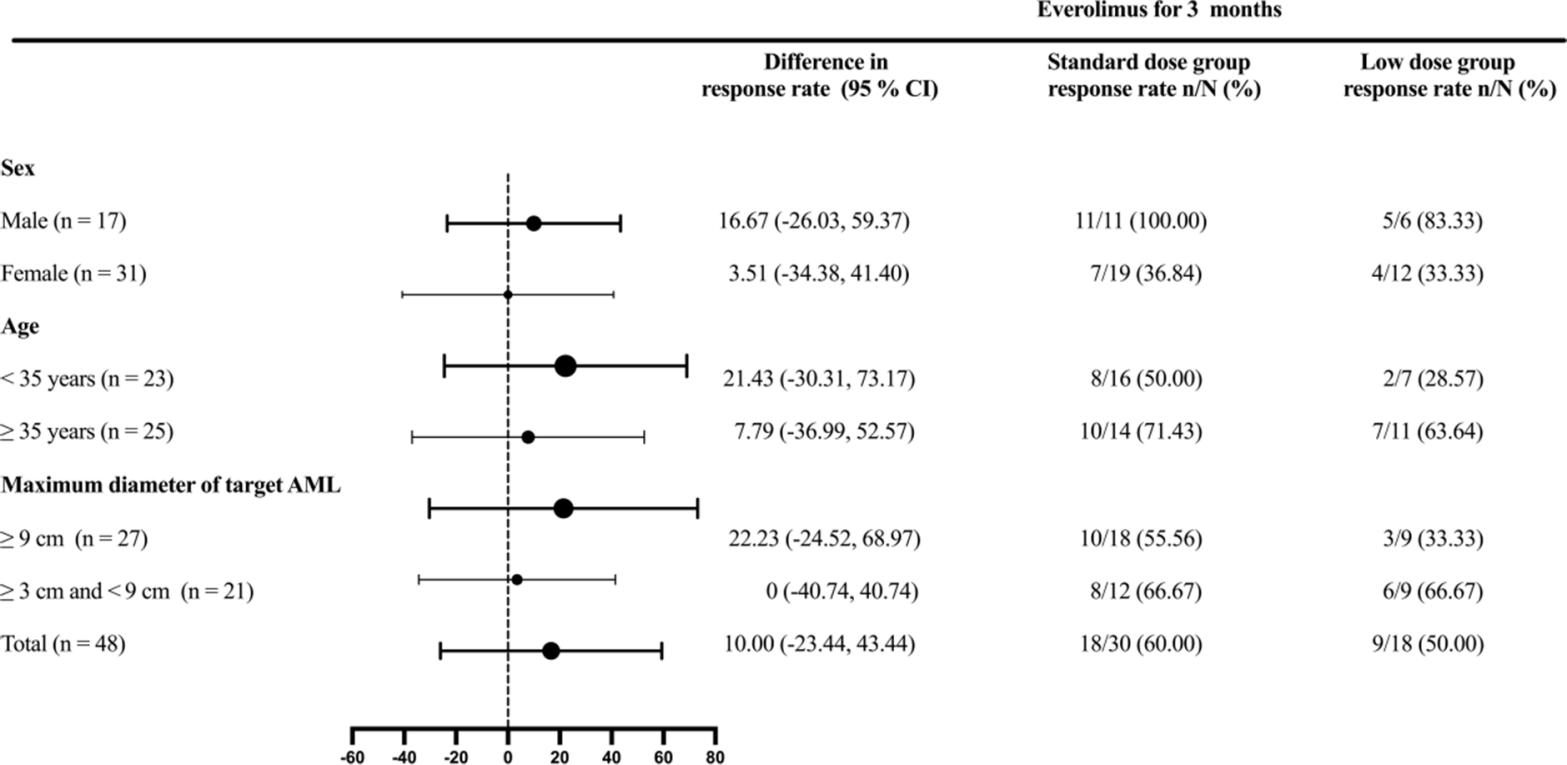
Forest plot of RAML response rates in patient subgroups. Area of each circle is proportional to the magnitude of the difference in the response rate. 95% CIs were obtained from exact unconditional confidence limits.

To investigate the potential of a low-dose everolimus regimen in the treatment of TSC-RAML and its role in improving long-term tolerability, we conducted a prospective cohort study comparing the efficacy of low- and standard-dosage everolimus. Both regimens produced rapid reductions in RAML tumour volume relative to baseline (**Supplementary Figure 4**). The proportions of patients achieving ≥50% reduction in target tumour volume were comparable between groups after 3 and 6 months of treatment (60% vs. 50% and 66.67% vs. 61.11%, respectively; **Table 4**), indicating no statistically significant difference in efficacy. Trough everolimus concentrations were monitored and maintained within the therapeutic target range of 5–15 ng/mL, consistent with current consensus recommendations. Subgroup analyses further demonstrated that tumour response rates (≥50% reduction vs. baseline) did not significantly differ between the two dosing regimens across sex, age, or baseline maximum RAML diameter (**Figure 4**). Collectively, these findings suggest that appropriately monitored low-dose everolimus may achieve comparable efficacy to standard dosing with improved safety and tolerability profiles.

**Table 4:** RAML volume response rate and partial response from baseline following treatment.

Time (months)	Standard dose group (10mg/day)	Low dose group (5mg/day)	P-Value
3 months			
Reduction from baseline (mean ± SD, %)	52.16 ± 21.68	45.05 ± 18.38	.251
No. PR/Total No. patients (%)	18/30 (60.00)	9/18 (50.00)	.499
6 months			
Reduction from baseline (mean ± SD, %)	58.34 ± 21.75	53.26 ± 20.26	.426
No. Of PR/Total No. of patients (%)	20/30 (66.67)	11/18 (61.11)	.697

PR: Partial response, defined as a ≥50% reduction in the volume of the largest target renal AML relative to baseline, with no evidence of angiomyolipoma progression.

A *P*-value < 0.05 was considered statistically significant.

### Adverse events associated with everolimus treatment

Patients in both groups were monitored for adverse events during 6 months of everolimus treatment. Most adverse events were mild to moderate (grade 1 or 2), with oral mucositis, headache, and nausea or vomiting being reported most commonly. Severe adverse events (grade 3 or 4) were rare—with six (20%) and two (11.11%) patients reporting grade 3 or 4 oral mucositis in the standard- and low-dosage groups, respectively. Other grade 3 or 4 events included irregular menstruation and acne-like skin lesions—only observed in the standard dose group (**Table 5**).

**Table 5:** Treatment-related main adverse events in 6 months.

Adverse event, n (%)	Standard dose group (10 mg/day)		Low dose group (5 mg/day)		P-Value
	All grade	Grade 3/4	All grade	Grade 3/4	All grade
Oral Mucositis	29 (96.67)	6 (20.00)	10 (55.56)	2 (11.11)	.002
Headache	13 (43.33)	0	4 (22.22)	0	.139
Irregular menstruation (female only)	8 (42.11)19	1 (3.33)	2 (16.67) 12	0	.240
Acne-like skin lesion	9 (30.00)	3 (10.00)	3 (16.67)	0	.491
Cough	7 (23.33)	0	3 (16.67)	0	.854
Upper respiratory tract infection	6 (20.00)	0	3 (16.67)	0	1.000
Non-infectious pneumonia	4(13.30)	0	2(11.10)	0	1.000,
Nausea/vomiting	11 (36.67)	0	4 (22.22)	0	.296
Fatigue	2 (6.67)	0	2 (11.11)	0	1.000
Urinary tract infection	1(3.33)	0	1(3.33)	0	1.000
Hemorrhage	0	0	0	0	–
Anemia	6 (20.00)	0	2 (11.11)	0	.689
High cholesterol	4 (13.33)	0	3 (16.67)	0	1.000

Adverse events such as cough, upper respiratory tract infection, fatigue, urinary tract infection, anemia, and high serum cholesterol were observed; however, their incidence did not differ significantly between the two groups. No cases of hemorrhage were observed, and no abnormalities were detected in other routinely measured laboratory parameters.

In general, all adverse events were managed through symptomatic treatment. Additionally, no patient required temporary everolimus discontinuation during the study period.

## Discussion

Currently, multiple clinical studies have established the significant therapeutic value of everolimus in the management of TSC. The pivotal phase III EXIST-2 trial demonstrated that after 6 months of treatment, 33/79 (42%; 95% CI, 31–53%) of patients achieved a ≥50% reduction in RAML volume, with a favorable safety and tolerability profile (34). Its long-term efficacy and safety were further consolidated in a subsequent 4-year follow-up study (22). In addition, the EXIST-1 and EXIST-3 trials confirmed the efficacy of everolimus in treating TSC-associated subependymal giant cell astrocytomas and focal seizures, respectively (35, 36). Although the efficacy of everolimus has been well validated, there is still limited evidence regarding tissue-specific responses and dose optimization strategies in the TSC-RAML cohort, particularly regarding the influence of tumour fat content. In our current multicenter study, 6 months of everolimus therapy achieved ≥50% tumour-volume reduction in 83% (60/72) of patients, followed by regrowth upon drug discontinuation. This 6−month treatment duration aligns with the 2024 ERKNet/ERA consensus recommendation that at least 6 months of follow−up is required to adequately evaluate the radiological response to mTORC1 inhibitors, as the major effect on angiomyolipoma volume occurs within the first 6–12 months (23).

Further analysis revealed that everolimus exhibited greater tumor-shrinking efficacy in fat-poor RAMLs than in fat-rich lesions. Finally, by comparing standard- and low-dose regimens, this study provides new insights for optimizing the clinical dosing strategy of everolimus in TSC-RAML. Importantly, this study provides evidence from a large, entirely Chinese patient cohort, thereby adding data on everolimus activity in an Asian population that has been underrepresented in previous trials.

In this study, the tumour volume of subjects returned to 62.5% of the baseline levels after 3 months after treatment discontinuation and returned to the baseline level after 6 months. This rebound phenomenon is also consistent with the consensus view that sustained control of TSC-associated angiomyolipoma may require individualized long-term treatment rather than short-course therapy alone. Hatano et al. reported that everolimus treatment remains reliable when discontinued after effective everolimus-based RAML control, and everolimus treatment should be reinitiated when RAML increases to 70% of the baseline levels (37). Although these phenomena indicate that long-term everolimus treatment may be required, prolonged everolimus use may lead to persistent adverse effects in patients and increases in their financial burden; this highlights the need to explore long-term treatment strategies. In a Taiwanese study,11 patients with TSC-RAML were treated with everolimus doses of 2.5–5 mg. Among the patients who remained on 2.5 mg, five demonstrated 10.6%–65.2% tumour volume reduction during follow-up, but three had a worse prognosis. In contrast, six patients who were escalated to a 5-mg dose achieved a 42.5%–70.6% tumour volume reduction without progression (38). Therefore, 5 mg of everolimus may be a reasonable individualized starting or maintenance dose for adult patients, consistent with the 2024 ERKNet/ERA consensus recommendation that 5 mg everolimus is a reasonable starting dose in adults with TSC. In the present study, we compared the short-term efficacy and safety of standard- and low-dosage everolimus and found no significant difference in the proportions of patients achieving ≥50% tumour volume reduction compared with baseline levels (66.67% vs. 61.11%). In the low-dosage group, trough everolimus concentrations were regularly monitored and maintained within the target therapeutic range of 5–15 ng/mL, consistent with current consensus recommendations. Notably, the low-dosage group exhibited significantly fewer cases of oral mucositis and incurred lower overall medical costs. These findings suggest that a fixed low-dose regimen, when guided by therapeutic drug monitoring to maintain adequate trough levels, may represent a viable and safer option for TSC-RAML management—offering improved tolerability, reduced economic burden, and potentially better long-term treatment adherence. It is crucial to emphasize that, due to the non-randomized design of this dose-comparison analysis and the inherent differences in baseline renal function and body weight between the two groups, the observed efficacy of the low-dose regimen should be regarded as exploratory and hypothesis-generating. The efficacy trend suggests that, guided by therapeutic drug monitoring, a 5 mg/day starting dose may be a candidate worthy of further investigation, but it should by no means be interpreted as proof of equivalence between the two dosing regimens. Confirming the non-inferiority of low-dose everolimus requires future large-scale, prospective, randomized controlled trials.

RAML is composed of abnormal neovascularisation, immature smooth muscle cells, and adipocytes at varying proportions. Studies on TSC-RAML have reported individual differences in responses to everolimus, with the treatment being ineffective in some cases. EXIST-2 and its extension study demonstrated that 50% of patients achieved a >55% reduction in RAML tumour volume after 6 months of everolimus treatment, highlighting the significant therapeutic effect of everolimus. However, some patients did not respond to the drug and experienced no reduction in tumour volume (34, 39). This may be because the therapeutic efficacy of mTOR inhibitors such as everolimus is closely associated with the tumour composition of TSC-RAML, particularly the fat content. RAML with a lower fat content tends to respond more effectively to treatment. In the present study, 57 patients with TSC-RAML were categorized into fat-rich (n = 30) and fat-poor (n = 27) groups based on tissue-specific attenuation thresholds, and changes in tumor volume and mean CT attenuation were analyzed before and after everolimus treatment. The fat-rich RAML group exhibited a weaker response to everolimus compared with the fat-poor group. Specifically, in the fat-rich group, two patients experienced an increase in tumor volume relative to baseline, and only 6.67% (2/30) achieved ≥30% reduction, while none reached ≥50% reduction. In contrast, all patients in the fat-poor RAML group showed tumor shrinkage, with 96.30% (26/27) achieving ≥30% or ≥50% volume reduction, indicating that tumor composition may represent an imaging biomarker for predicting everolimus responsiveness. The biological basis underlying this observation is likely related to the differential dependence of individual TSC-RAML tissue compartments on mTOR signaling. Recent spatial transcriptomic profiling of TSC-associated AML demonstrated triphasic tumor architecture composed of vascular, smooth muscle, and adipose compartments, with mild upregulation of MTOR, MLST8, and RPTOR consistent with mTORC1 hyperactivation, as well as region-specific enrichment of angiogenesis, extracellular matrix remodeling, and lipid metabolic (PPAR signaling) pathways (40). A prior multi-omics study incorporating bulk and single-cell RNA sequencing further showed marked microenvironmental heterogeneity in TSC-RAML, including tumor-like cells, fibroblasts, and monocyte/macrophage infiltration, supporting the presence of distinct cellular programs within these lesions (41). Consequently, everolimus may preferentially target the mTOR-driven smooth muscle and vascular compartments, leading to substantial volume reduction in fat-poor lesions; in fat-rich AMLs, by contrast, the relative predominance of mature adipocytes and lipid-storage programs may mask the partial response of the vascular and smooth muscle components, resulting in more limited overall shrinkage (42). Taken together, these findings provide a plausible biological framework for our observation that fat-poor lesions exhibit greater volume reduction after everolimus therapy; however, this interpretation remains inferential and should be confirmed by future histopathologic and spatial molecular validation studies.

Importantly, these observations also have potential clinical implications. Because CT attenuation reflects the relative proportions of adipose and non-adipose tissue within TSC-RAML, baseline tumor density may serve as a readily accessible imaging biomarker for predicting responsiveness to mTOR inhibition and facilitating individualized treatment strategies. Future studies integrating quantitative imaging, spatial transcriptomics, and histopathological validation will be valuable for establishing a biologically informed imaging classification system for therapeutic decision-making. Nevertheless, several limitations should be acknowledged. Our study included only adult patients and excluded lesions with intermediate CT attenuation values (−10 to 30 HU), which may limit the generalizability of the proposed classification and warrants further validation in broader patient populations.

Notably, the adverse events associated with everolimus treatment in this study were generally consistent with those reported in previous studies on everolimus in TSC settings (34, 43, 44). Most adverse events had a severity grade of 1 or 2, with oral mucositis, headache, and nausea or vomiting being observed most frequently. This result highlights the importance of educating patients on oral care to reduce stomatitis incidence. Despite the occurrence of the side effects, no patients in this study discontinued treatment or required a dose reduction due to adverse events. Rimon et al. developed a semiquantitative vascular grading system for RAML and reported that TSC-RAML lesions with more abundant vascular tissue are more prone to hemorrhage (45). However, no hemorrhagic events were observed in this study, suggesting that everolimus may have a protective effect against hemorrhage. Unfortunately, we did not further analyse the changes in tumour vascular composition before and after treatment.

Several limitations of this study should be acknowledged. First and foremost, the dose-comparison analysis was based on a nonrandomized, open-label design. Although the criteria for dose selection were informed by previously published clinical protocols (25) and aligned with the 2024 ERKNet/ERA consensus recommendation that 5 mg/day is a reasonable starting dose, the nonrandomized nature of treatment allocation may have introduced selection bias. Patients assigned to the low-dose group tended to have renal dysfunction or lower body weight, potentially making the groups prognostically dissimilar at baseline. Second, the unequal group sizes in the dosing cohort (n = 30 standard-dose vs. n = 18 low-dose) further limit the statistical power and precision of the comparative estimates. Third, treating clinicians were aware of the administered dose, introducing potential ascertainment bias in the reporting of subjective adverse events. Fourth, the fat-composition analysis in this study was based on a dichotomous comparison (fat-rich vs. fat-poor) and excluded lesions with intermediate HU values between −10 and 30. In our cohort, such lesions accounted for approximately 26.0% of patients with technically eligible imaging, meaning our predictive model is currently most applicable to tumors at the extremes of the fat-content spectrum. While this exclusion enhanced intergroup contrast and reproducibility, it does limit the direct generalizability of our findings to unselected, full-spectrum TSC-RAML patients. Future studies should explore whether everolimus efficacy varies along a continuous gradient of HU values and develop predictive models that can incorporate intermediate-density lesions to improve clinical utility. In light of these limitations, the observed comparable efficacy between the standard- and low-dose regimens should be interpreted as hypothesis-generating, and the predictive value of fat content requires validation in broader populations. The findings of the present study, taken together with the landmark EXIST-2 trial (34), which demonstrated the efficacy of 10 mg/day everolimus in a randomized controlled setting, support the need for future large-scale randomized controlled trials to validate the noninferiority of low-dose everolimus in patients with TSC-RAML.

## Conclusions

In summary, everolimus effectively reduces tumour size and appears to exhibit greater activity in fat-poor RAMLs. The exploratory findings of this study provide preliminary support for individualized dosing strategies and warrant further investigation of low-dose everolimus in selected patients. However, randomized controlled studies are required before low-dose regimens can be recommended for routine clinical practice.

## Data Availability

The original contributions presented in the study are included in the article/**Supplementary Material**. Further inquiries can be directed to the corresponding authors.
