# Supplementary material for: Tumor fat composition predicts response to everolimus and supports low-dose therapy in TSC-associated renal angiomyolipomas: a multicenter prospective study
Source: Front Oncol. 2026 Jul 22;16:1893155. doi: 10.3389/fonc.2026.1893155 (PMC13437317; doi:10.3389/fonc.2026.1893155)
Supplement: Supplementary file 1 [file DataSheet1.docx]

**SUPPLEMENTAL INFORMATION**

**Tumor fat composition predicts response to everolimus and supports low-dose therapy in TSC-associated renal angiomyolipomas: a multicenter prospective study**

*Ke* *Gao,* *^†,1^* *Yongfei Duan,* *^† ,^**^1^* *Biqi Ren,**^2^* *Boxin Guo,**^1^* *Ruiping Su,^1^* *Hao Gu,^1^ Fulin Chen,**^3^ Chi Zhang,^1^**Guojun Wu,^*,^**^1^* *Jianxin Ni^*, 1^*

^1^ Department of Urology, Xi’an People’s Hospital (Xi’an Fourth Hospital), School of Life Sciences and Medicine, Northwest University, Xi’an, 710199, China

^2^ Department of Scientific, Xi’an People’s Hospital (Xi’an Fourth Hospital), School of Life Sciences and Medicine, Northwest University, Xi’an, 710199, China

^3^ Laboratory of Tissue Engineering, College of Life Sciences, Northwest University, Xi'an 710069 Shaanxi, China

**^*^** **These authors jointly supervised this work and share corresponding authorship**

**Guojun Wu,** Department of Urology, Affiliated Xi’an Peoples Hospital(Xi’an Fourth. Hospital) of Northwest University, Hang Tian East Road, No. 155, Xi’an 710199, China.

**Email:** [wuguojun@med.nwu.edu.cn](mailto:wuguojun@med.nwu.edu.cn);

**Jianxin Ni**, Department of Urology, Affiliated Xi’an Peoples Hospital(Xi’an Fourth. Hospital) of Northwest University, Hang Tian East Road, No. 155, Xi’an 710199, China.

**Email:** nijianxin@vip.sina.com

*Ke Gao1†,* *Yongfei Duan 2†* These authors share first authorship.

**Supplemental figures and table**

**Figure S1.** Common clinical features of TSC patients. a. Head CT scan showing subependymal nodules; b. Abdominal CT scan showing typical renal angiomyolipomas; c-g. Classic dermatological manifestations: hypomelanotic macules, shagreen patch, subungual fibroma of the nail, facial angiofibroma.

**Figure S2.** Representative imaging of RAML in TSC patient treated with everolimus at different time points.

**Figure S3.** Change in RAML volume in patients following everolimus discontinuation. a. Volume reduction ratio from baseline during everolimus treatment and after its discontinuation. *****p*<.0001 by One-way ANOVA. b. Best percentage change from baseline in renal AML volume at 3 and 6 months following everolimus discontinuation.

**Figure S4.** Best percentage change from baseline in the volume of the largest target renal AML treated with varying doses of everolimus.

**Table S1. Pearson correlation analysis of factors associated with ≥50% reduction in largest target renal AML volume after everolimus treatment.**

| **Variables** | **An at-least-50% tumor volume reduction** | | |
| --- | --- | --- | --- |
|  | Pearson correlation coefficient | Statistical significance (2-sided ) | N |
| **Age** | -0.165 | .221 | 57 |
| **Fat** | 0.965^*^ | <.001 | 57 |
| **Sex** | 0.108 | .423 | 57 |

* There was a significant association at 0.01 level (2-sided).
